# Kinematic Analyses of the Thumb during Simulated Posteroanterior Glide Mobilization

**DOI:** 10.1371/journal.pone.0161624

**Published:** 2016-09-01

**Authors:** Meng-Tzu Hu, Ar-Tyan Hsu, Fong-Chin Su

**Affiliations:** 1 Institute of Biomedical Engineering, National Cheng Kung University, Tainan, Taiwan; 2 Department of Physical Therapy, College of Medicine, National Cheng Kung University, Tainan, Taiwan; 3 Institute of Allied Health Sciences, College of Medicine, National Cheng Kung University, Tainan, Taiwan; 4 Medical Device Innovation Center, National Cheng Kung University, Tainan, Taiwan; 5 Department of Physical Therapy, Tzu Hui Institute of Technology, Ping Tung, Taiwan; Brown University, UNITED STATES

## Abstract

**Objective:**

Thumb problems are common in some health professionals such as physical therapists. The purpose of this case-control study is to investigate the influence of clinical experience and different mobilization techniques on the kinematics of the thumb.

**Methods:**

Twenty-three participants without exposure to manual techniques (the Novice Group) and fifteen physical therapists with at least 3 years of orthopedic experience (the Experienced Group) participated. The kinematics of the thumb while performing 3 different simulated posteroanterior (PA) glide mobilization techniques on a load cell was monitored. These 3 techniques were: 1) unsupported, 2) with digital support and 3) with thumb interphalangeal joint supported by the index finger. The amount of forces exerted were 25% to 100% of maximum effort at 25% increments. The main effects of experience and technique on thumb kinematics were assessed.

**Results:**

Both experience and technique had main effects on the flexion/extension angles of the thumb joints. Experienced participants assumed a more flexed position at the carpometacarpal (CMC) joint, and the novice participants performed with angles closer to the neutral position (F = 7.593, p = 0.010). Participants’ metacarpophalangeal (MCP) joints were in a more flexed position while performing PA glide with thumb interphalangeal (IP) joint supported by the index as compared to the other two techniques (p < .001).

**Conclusions:**

Negative correlations were generally obtained between the sagittal plane angles of adjacent thumb joints during mobilization/manipulation. Therapists are recommended to treat patient with more stable PA glide mobilization techniques, such as PA glide with thumb interphalangeal joint supported by the index finger, to prevent potential mobilization-related thumb disorders.

## Introduction

The specific anatomical structural arrangement of the thumb provides the human hand with the unique capacity of a powerful grip as well as precision object manipulation [1]. Manual therapy procedures, such as joint mobilization, manipulation and soft tissue techniques frequently involve complicated and strenuous loading of the thumb joints. These techniques are used by several health professionals such as physical therapists, occupational therapists and massage practitioners. The Posteroanterior (PA) glide mobilization techniques described by Maitland [2] are commonly used by physical therapists to increase spinal mobility with sustained or rhythmic oscillatory forces. These forces are imposed on the spine by the body weight through the stabilized thumb and the rest of the upper extremity [2]. During applying forces, the positions of both interphalangeal (IP) and metacarpophalangeal (MCP) joints are encouraged to be in a neutral position with the thumbs in contact with each other. Such an alignment is capable of transmitting force and reduces the tendency of a zigzag collapse of the thumb’s multi-articular chain [3]. Few studies have investigated the kinematics and kinetics of a therapist’s thumb while performing such activities [4–7]. Buckingham et al. [4] observed that the thumb quickly deviated from the recommended position while physiotherapy students performed PA mobilizations with a clinically-representative maximum force of 122.86 N. The authors suspected that the deviation of the thumb position during large force application might explain the complaints made by young physiotherapy graduates found in Cromie’s study [8]. Factors, such as individual experience and different techniques used to complete the task may result in differential upper extremity kinematics [9]. Fattapposta et al. [10] suggested that long-term practice results in a better coordinated neuromuscular control and movement pattern. Through practice, variability of trajectory (velocity and position) has been shown to decrease and become more consistent [11, 12]. In addition, the degree of joint laxity has been identified as a factor influencing musculoskeletal injuries [13]. In subjects with hypermobility syndrome, proprioceptive deficiency may play a part in thumb kinematics as it has been reported in the proximal interphalangeal joint of the index [14]. These factors may potentially contribute to thumb kinematics and work-related injury in physical therapists. Thumb alignment while performing PA glide mobilization was included in an observational study in a conference performed on an artificial device with self-reported thumb pain that was only focused on the positions of the IP and MCP joints [15]. There is a lack of data quantifying the attributes of the kinematics of the thumb while executing mobilization. Video-based motion analysis systems have been applied in quantifying the complex kinematics of the thumb during activities of daily living [16–20]. A better understanding of thumb alignment during PA glide mobilization should provide a foundation for future studies for the relations among the potential risks related to laxity, thumb position and mobilization techniques on clinical applications of such manual techniques. Furthermore, there may be practical benefits of providing protective strategies against occupation-related disorders involving the thumb. Therefore, the purposes of this study were to investigate the influence of clinical experience, joint laxity features and different PA glide mobilization techniques on thumb kinematics while performing PA glide mobilization with the thumb in a group of physical therapists and their novice counterparts. We hypothesized that the alignment of the thumb would differ with clinical experience, joint laxity features, and different PA glide techniques.

## Materials and Methods

### Participants

A total of 38 participants were included in this study. The Novice Group included 23 healthy young participants who had no prior exposure to manual therapy. The Experienced Group included 15 healthy participants who had at least 3 years of clinical experience in orthopedic physical therapy. Participants with any history of musculoskeletal or neurological disorders were excluded. All participants were right-hand dominant. No pain involving the use of thumb such as PA glide mobilization was reported.

### Instrumentation

**Fig 1:**
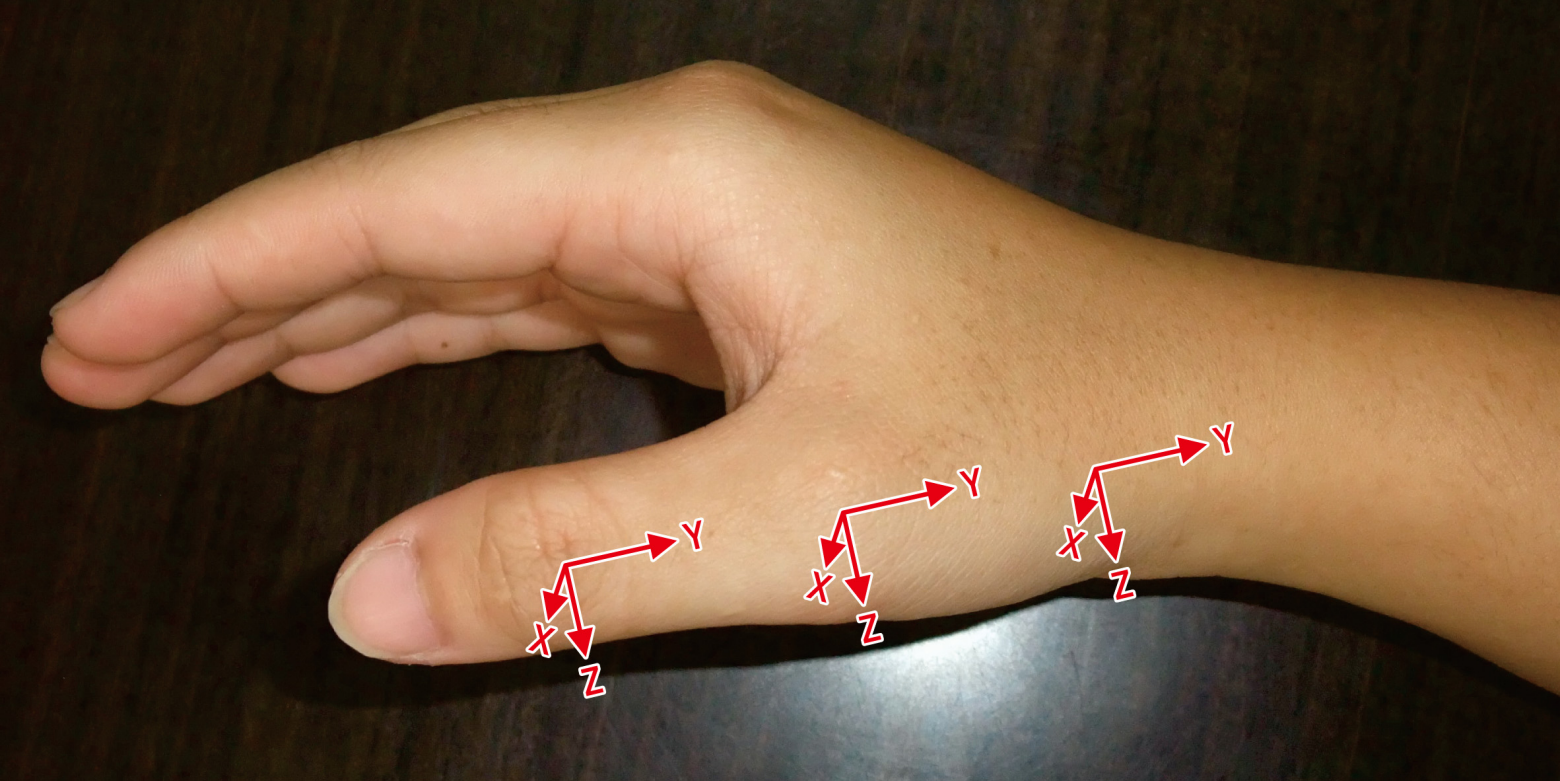
The local coordinate systems of the right thumb segments were shown. X axis = dorsal/ volar axis; Y axis = distal/proximal axis; Z axis = ulnar/radial axis

The three-dimensional kinematic data of the thumb were acquired by a six-camera VICON motion analysis system at a frame rate of 200 Hz. The motion analysis system tracked the movements of markers placed on specific bony landmarks to compute the angles of each joint during the experimental procedure. The coordinate system of each thumb segment was defined in accordance with the International Society of Biomechanics convention [21]. Positive angles for flexion occurred about the ulnar-radial axis (Z-axis), pronation occurred about the distal-proximal axis (Y-axis), and adduction occurred about the dorsal-volar axis (X-axis) (Fig 1). Forces and moments applied through the thumb were registered by a 6-axis load cell at a 50Hz sampling rate. The load cell was fixed on top of an L-shaped plate rigidly clamped onto a table. The vertical side of the L-plate paralleled one side of the load cell and was the same height as the load cell for the purpose of supporting the rest of the digits during the mobilization task when needed.

### Experimental Procedures

Each participant read and signed an informed consent form and answered a questionnaire concerning his/her history of pain and disabilities relating to the thumb and hand before testing. The Beighton score [22] of each participant was recorded. Three joint laxity levels were categorized according to their Beighton scores: (1) normal individuals, with none or only one positive Beighton criterion; (2) lax individuals, with two or three positive Beighton criteria; (3) hyperlax individuals, fulfilled four or more Beighton criteria. The experimental protocol was approved by the Institutional Review Board of National Cheng Kung University Hospital.

Four sets of retro-reflective marker clusters were place on participant’s hand. Each consisted of a T shaped plastic plate and three 4-mm markers. Two markers from each marker cluster were aligned with the long axis of each segment of the thumb and the distal radius (radial styloid process). Markers were also placed on the medial and lateral aspect of the IP and MCP joints to define the centers of the IP and MCP joints. To reduce inter-rater error, all markers were placed with the thumb in neutral position and were identified by a physiotherapist with 5 years of experience in orthopedic physical therapy. The hand was then placed on a rigid splint with the wrist and the palm maintained in a fixed position to record the neutral position of the thumb. In this position, the first metacarpal was aligned with the proximal and distal phalangeal bones, and the thumb was in the mid-range of the abduction and flexion. The alignment of the thumb paralleled the axis of the 2^nd^ metacarpal.

Small arc thumb movements consisting of adduction/abduction, flexion/extension, and circumduction were also recorded with the VICON motion analysis system while the wrist and hand were held in this position for the purpose of estimating the center of rotation of the CMC joint.

**Fig 2:**
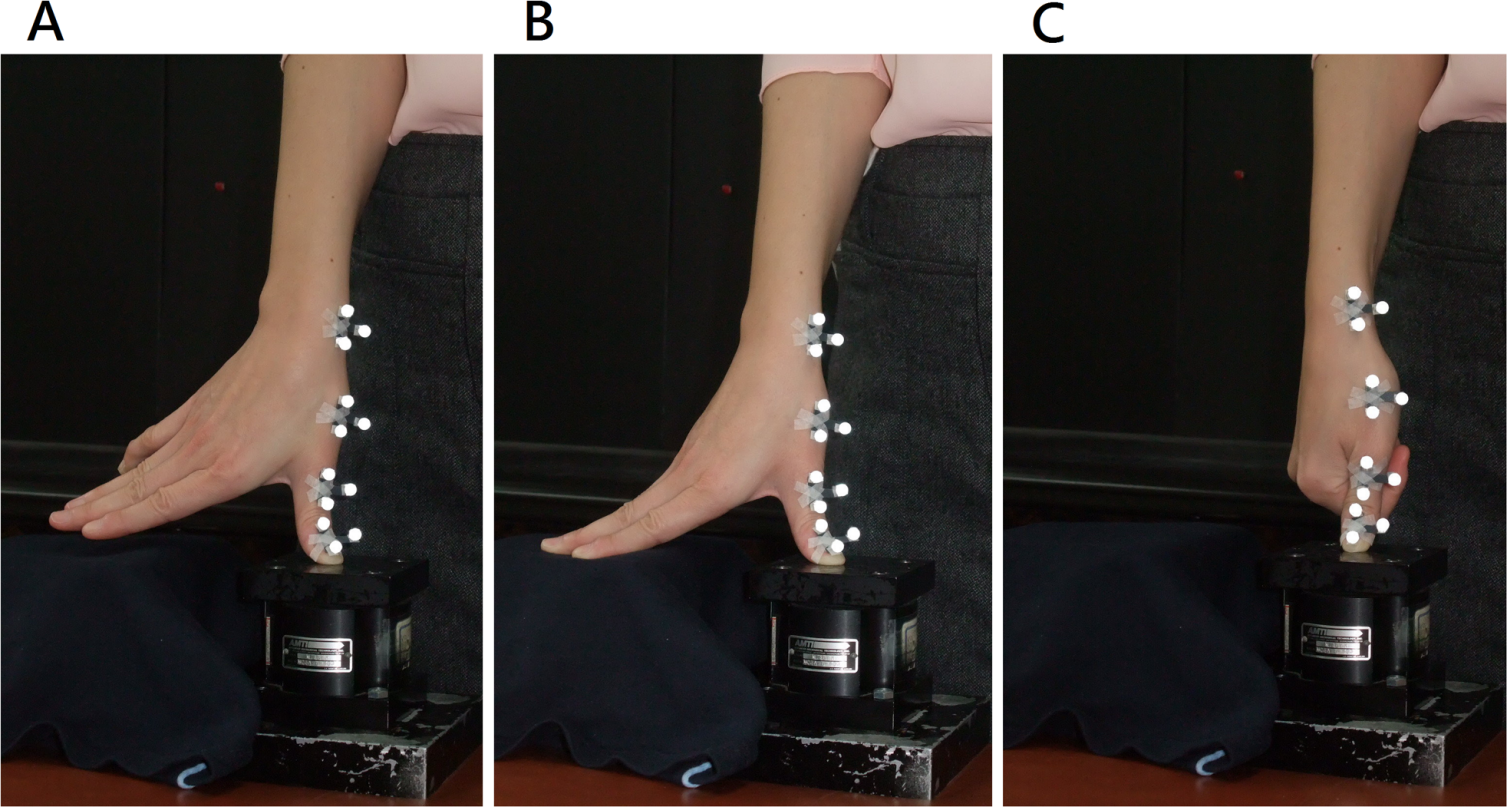
Three PA glide techniques were performed on the load cell. (A) T1, unsupported PA glide; (B) T2, digits supported PA glide; (C) T3, PA glide with IP joint supported by the index finger.

The following PA glide techniques were tested in a random order on the top plate of the 6-axis load cell while the VICON system tracked the trajectory of the thumb movements (Fig 2).

1. Unsupported PA glide (T1): The PA glide of the thumb was performed with the rest of the digits unsupported.
2. Digits supported PA glide (T2): The PA glide of the thumb was performed with the rest of the digits supported on the side of the L-plate.
3. PA glide with support by the index at the palmar aspect of the interphalangeal joint of the thumb (T3).

For the purpose of the study design, the kinematics of the thumb was analyzed at the objective constant force level. The participants were asked to exert a maximum force on the force plate and to try to maintain the IP and MCP joints in neutral position while performing each PA glide technique first. The recorded force was set to 100%. The participants were asked to view the monitor that displayed the target force level from 0% to 100% at 25% increments and the real time force output in two separate columns. The participants were then instructed to exert at a force level matching each of the target force levels displayed on the monitor as closely as possible for a period of five seconds. Verbal feedback with regard to the accuracy of the target force was also provided. The trajectory of each marker and the thumb tip force responses were recorded simultaneously.

### Data and Statistical Analyses

The raw kinematic (200Hz sampling rate) and force data (50Hz sampling rate) were filtered with a third-order Butterworth low-pass filter at 5 Hz. The neutral position of the thumb joint recorded with the brace in place was assigned as the resting position. The angle of the MCP and IP joints were computed. The T1 data from one of the experienced participants was excluded as an outlier due to extreme values in the range of the IP joint. The data was analyzed using the SPSS for Windows v.14.0. A mixed model 3 way (group x2, technique x3, joint laxity feature x3) ANOVA with repeated measures was used to analyze the angles of each thumb joint. The Bonfferroni *post-hoc* test was applied when significant differences were revealed by ANOVA. A Pearson correlation was used to examine the angle-angle relationship among the sagittal plane movements of the IP, MCP and CMC joints at different force levels. The alpha value was set at 0.05. A post hoc power analysis was also performed on each variable with G*Power 3.1 [23].

## Results

There was no difference found between the Novice and Experienced Group in regard to the basic demographic data except age and clinical manual experience (Table 1). The joint laxity features of two groups in accordance with the Beighton scores were also showed in Table 1. Table 2 showed the averaged joint angles during the application of the three mobilization techniques in both groups while executing their maximal effort. At the MCP joint, there was a main effect of technique on the sagittal (flexion/extension) angle, and no interaction was found. The post hoc analyses showed that the MCP joint in T3 was in a more flexed position than that of the other two techniques [Table 2, Novice: T3 (7.904°± 20.648°) > T1 (-10.186°± 26.470°); Experienced: T3 (11.979°± 18.183°) > T1 (-18.679°± 24.014°), 95% CI = 12.341 to 37.324, p < .001; Novice: T3 (7.904°± 20.648°) > T2 (-21.803°± 20.002°); Experienced: T3 (11.979°± 18.183°) > T2 (-14.302°± 21.937°), 95% CI = 17.232 to 39.869, p < .001]. At the CMC joint, a significant group main effect and no interaction was found on its sagittal angle. The Experienced Group assumed a more flexed position at the CMC joint, and the Novice Group had angles closer to the neutral position (Table 2). At the IP joint, no interaction and no main effect of technique (F = 2.175, p = 0.122) or group (F = 2.753, p = 0.107) was found on the flexion/extension angles. No main effect of joint laxity was found on all thumb flexion/extension angles.

**Table 1:** Demographic Description of the participants.

Basic data	Novice Group (N = 23)	Experienced Group (N = 15)
Age (years)*	21.1 (2.2)	28.0 (2.1)
Gender (male / female)	12 / 11	5 / 10
Height (cm)	168.2 (7.3)	162.9 (8.0)
Weight (kg)	61.5 (17.0)	56.9 (12.1)
BMI (kg/m^2^)	21.5 (4.3)	21.3 (3.3)
Clinical experience (years)*	0.0 (0.0)	4.3 (1.4)
Beighton score		
Normal	6 (26.1%)	8 (53.3%)
Lax	7 (30.4%)	3 (20.0%)
Hyperlax	10 (43.5%)	4 (26.7%)

NOTE. Values are mean (SD) or as otherwise indicated. Normal: with a Beighton score of 0 or 1; Lax: with a Beighton score of 2 or 3; Hyperlax: with a Beighton score of 4 or more. No significant difference between two groups in Beighton score with χ^2^ = 2.902; p = 0.234

* p<0.01

**Table 2:** Descriptive statistics of thumb flexion/extension joint angles (degrees) while performing three techniques at maximal force.

		Novice Group (N = 23)	Experienced Group (N = 15)	Technique effect
IP				
	T1	-6.073 (28.313)	-19.800 (29.306)	F = 2.175 p = 0.122
	T2	0.817 (28.834)	-16.441 (33.256)	
	T3	-10.453 (21.931)	-25.239 (21.310)	
Group effect		F = 2.753 p = 0.107		
MCP				
	T1	-10.186 (26.470)	-18.679 (24.014)	F = 15.806 p < 0.001^†^
	T2	-21.830 (20.002)	-14.302 (21.937)	
	T3	7.904 (20.648)	11.979 (18.183)	
Group effect		F = 0.040 p = 0.844		
CMC				
	T1	-1.112 (13.647)	10.121 (11.416)	F = 0.089 p = 0.915
	T2	-0.089 (13.330)	5.978 (15.604)	
	T3	0.723 (10.457)	8.133 (13.156)	
Group effect		F = 7.593 p = 0.010*		

NOTE. The values of each kinematic variable are expressed as means (SD). A positive angle indicates flexion, and a negative angle indicates extension. Differences are listed (1) between Experienced Group and Novice Group, (2) among the three mobilization techniques.

Abbreviations: T1, unsupported PA glide; T2, PA glide with digital supported; T3, PA glide with IP joint supported by the index.

* p<0.05

^†^ p<0.01

**Fig 3:**
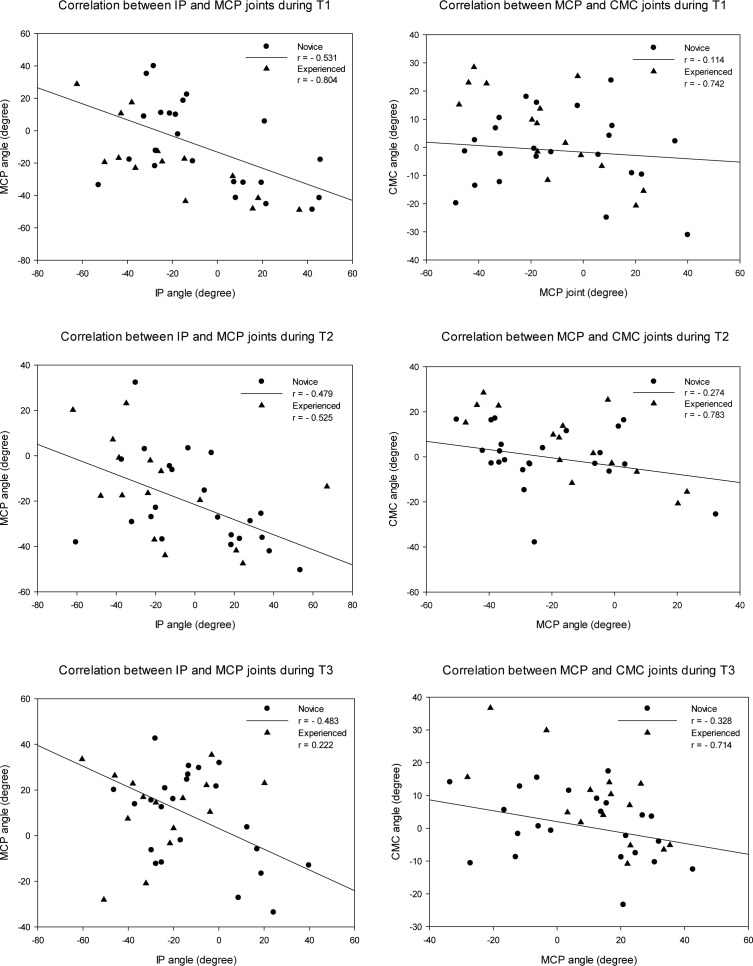
Correlations of the thumb IP and MCP joints as well as the MCP and CMC joints while performing three PA glide techniques with maximum effort in both groups. The positive angle indicates flexion, and the negative angle indicates extension.

In the Novice Group, most of the flexion/extension angles at the MCP joint were negatively correlated with those of the IP joint while executing all techniques at all force levels, especially at higher force levels (r = -0.282 ~ -0.659, p < .05). In the Experienced Group, negative correlations were obtained between the MCP and CMC joints while performing all three techniques at all force levels (r = -0.516 ~ -0.860, p < .05). Fig 3 shows generally negative correlations between adjacent joints while performing PA glide mobilization with maximal force in both groups. Table 3 reveals various thumb patterns in accordance with the position of the IP, MCP and CMC joints (IP-MCP-CMC) in these two groups. The Novice Group had greater variability in thumb alignment patterns. The most common pattern in the Experienced Group was EEF (Extension-Extension-Flexion) while performing T1 and T2. In the case of T3, the IP joint in extension and the MCP joint in flexion (EFE or EFF) were the most common alignments in both groups.

**Table 3:** Number of thumbs (n) of the thumb alignments which were categorized according to the positions of the IP, MCP and CMC joints (IP-MCP-CMC) during maximal performance of PA glide mobilization in both groups.

IP-MCP-CMC	T1		T2		T3	
	Novice (N = 23)	Experienced (N = 14)	Novice (N = 23)	Experienced (N = 15)	Novice (N = 23)	Experienced (N = 15)
E-E-F	4	7	3	6	2	3
E-E-E	2		5	2	2	
E-F-F	4	1	1		6	7
E-F-E	4	2	2	3	6	4
F-E-F	2	4	6	3	3	
F-E-E	6		5	1	2	
F-F-F			1		1	
F-F-E	1				1	1

NOTE. The values of each thumb alignment are the numbers of each group.

Abbreviations: T1, unsupported PA glide; T2, PA glide with digital supported; T3, PA glide with IP joint supported by the index; E, extension; F, flexion.

## Discussion

In this study, the 1^st^ CMC of the experienced subjects assumed a more flexed position compared with that of the Novice Group, who adopted a more ‘neutral’ position (Table 2). Zancolli [24] mentioned that in the opposition phase and distal pinch grip (abduction and flexion), the articular contact between the trapezium and metacarpal crests is maximal and relatively stable. Therefore, the experienced participants in this study positioned their CMC joint in a more stable situation. Different bony configurations, tissue constraints, the biomechanical mechanisms of tasks and neuromuscular control may have collectively influenced the force direction vectors. While performing downward forces during T1 and T2, half of the experienced participants revealed extension alignment (Table 3) and statistically revealed a more extended position at both the IP and MCP joints (>14° extension). For the purpose of directing forces from the upper body to the thumb tip, the CMC joint had to be in a more flexed position to transmit forces to the tip. However, no proper CMC posture while performing PA glide mobilization has been proposed in the literature. It remains unclear why and how different thumb positions were adopted by experienced and novice participants. More researches on these aspects of the joint mobilization are needed in the future.

With few exceptions, negative correlations between the sagittal plane angles of adjacent joints (IP vs MCP, MCP vs CMC, Fig 3) were found in both groups. Such findings indicate that when the IP was being pushed into extension by the reaction force, the MCP joint was forced into flexion, and the CMC joint was forced into extension. This inverse relationship might have been initiated by the contact made between the thumb pad and the targeted bony landmark, in our case, the top surface of the force plate. The offset arrangement between the nail and the distal phalangeal bone (and thumb pad) prevents a straight vertically downward contact. Therefore, the contact is usually made at the distal portion of the thumb pad, and a dorsal-proximally directed reaction force is created thus forcing the distal phalanx into extension. The need for maintaining the stability of the IP joint sets off a serial reaction along the kinetic chain of the thumb. It was interesting to note that the Experienced Group in the present study exhibited significant negative correlations between the IP and MCP joints with the exception of T3 and between the MCP and CMC joints in all 3 techniques. However, the Novice Group exhibited significant negative correlations only between the IP and MCP joints (Fig 3). This may indicate that different strategies were adopted for maintaining thumb stability between these two groups. Novice participants appeared to respond to tip forces by controlling the IP joint without paying proper attention to the proximal joints while experienced participants controlled both the IP and CMC joints during the process of directing force to the target. Li et al. [17] observed interjoint coordination, which was coordination among multiple thumb joints in a specific thumb movement direction. This interjoint coordination may be attributable to the extrinsic muscles and torques created by the tensioned muscles on the joints it crosses [17]. Although tasks performed in this study were different from Li’s study [17], we suspected that the negative correlation between adjacent joints was a result of interjoint coordination for the PA glide technique requirements. Due to the different levels of experience, each group likely adapted to different control patterns in order to direct force more efficiently.

While performing T1 and T2, nearly half of the experienced participants revealed a kinematic alignment of the MCP extension and CMC flexion (EEF and FEF in Table 3). Moulton et al. [25] suggested that hyperextension at the MCP joint will result in reciprocal flexion of the metacarpal and dorsal subluxation of the CMC joint. This situation concentrates the stress on the palmar articular surface and accelerates the development of osteoarthritis of the CMC joint [26, 27]. Although the Experienced Group adopted a more congruent (flexed) CMC joint position for more efficient thumb tip force generation, the hyperextension of the MCP joint (MCP >10° extension) contributed to the concentrated stress on the CMC joint while performing T1 and T2. Therefore, it is not surprising that thumb pain is one of the most common job-related disorders in physiotherapists [28–30].

In the present study, the joint laxity of the participants did not play a significant role in the participant’s adoption of the thumb position during PA glide mobilization. The technique characteristics may thus play a more important role. While performing T3, the IP joint is supported by the index, and more intrinsic muscles (adductor pollicis and first dorsal interosseus) are activated [7]. The adductor pollicis pulls at the base of the proximal phalanx [31], and this, combined with the reaction torque from the tip force, may contribute to more flexion tendency of the MCP joint in T3 (Table 2). In Moulton’s study [25], flexion of the MCP joint was found to effectively unload the higher forces at the palmar compartment of the CMC joint, which is correlated with progressive osteoarthritis. Moreover, more activity of the first dorsal interosseus provides distally and ulnarly directed pull to the first metacarpal for the purpose of preventing a radial and dorsal subluxation force from the adductors and flexors [31] and, in the meantime, reduces the compressions force at the CMC joint. The relative errors in thumb tip force while performing T3 were also smallest [7]. Thus, T3 is more stable and is a more mechanically advantageous technique when compared with the other two techniques.

The post hoc power analyses revealed a large effect size (>.4) and power (>.80) [32] in the main effects of experience (group) and technique. However, limited statistical power may have played a role in detecting the differences in joint laxity. Larger sample sizes are needed in the future for detecting the difference on the joint laxity variable.

### Study Limitations

Some limitations are inherent in this study. First, most PA glide techniques employed for the human spine region are usually applied bimanually while only the right dominant thumb was tested against a force plate in the present study. The force applied through the thumb on the force plate may cause discomfort, which may limit force production due to the lack of cushion provided by the skin and subcutaneous tissue. Second, the CMC joint kinematics in this study was referred to as the relative motion of the first metacarpal and the radius. As a result, it presented the combined movement of the radio-carpal and carpometacarpal joints. Third, this study excluded therapists with thumb pain. Its purpose was to avoid adaptive responses induced by pain while executing PA glide mobilization. Consequently, the present study could not establish the relationship between thumb pain and PA glide mobilization. Moreover, most therapists without pain included were with normal joint laxity (53.3%) which is different from that of the novice participants (26.1%). This is also a limitation for detecting the effect of joint laxity. Future research studies are needed to determine the relationship between work-related thumb pain and its alignment/technique related to mobilization. Lastly, although gender did not exhibit a main effect on the kinematics of the CMC joint during three isometric functional tasks [33], female dominance may be a limitation in the Experienced Group in this study.

## Conclusions

While performing PA glide mobilization in either stable or unstable positions, experienced participants kept the CMC joint in a more flexed position for the generation of thumb tip force while novice participants directed the tip force by controlling the IP joint. In addition, the finding also suggests that therapists should be encouraged to employ more stable mobilization techniques, such as a PA glide with the thumb interphalangeal (IP) joint supported by the index (T3), to position the MCP joint in a more flexed position and to avoid mechanically disadvantageous hyperextended positions.

## Supporting Information

S1 Checklist This is the checklist of the present study. (PDF)
