# Supplementary material for: Kinematic Analyses of the Thumb during Simulated Posteroanterior Glide Mobilization
Source: PLoS One. 2016 Sep 1;11(9):e0161624. doi: 10.1371/journal.pone.0161624 (PMC5008622; doi:10.1371/journal.pone.0161624)
Supplement: S1 Checklist — (PDF) [file pone.0161624.s001.pdf]

STROBE Statement—Checklist of items that should be included in reports of ***case-control studies***

	Item No	Recommendation
**Title and abstract**	1	(*a*) Indicate the study's design with a commonly used term in the title or the abstract line 22
		(*b*) Provide in the abstract an informative and balanced summary of what was done and what was found lines 24-41
**Introduction**		
Background/rationale	2	Explain the scientific background and rationale for the investigation being reported lines 44-75
Objectives	3	State specific objectives, including any prespecified hypotheses lines 82-86
**Methods**		
Study design	4	Present key elements of study design early in the paper lines 91-97
Setting	5	Describe the setting, locations, and relevant dates, including periods of recruitment, exposure, follow-up, and data collection lines 122-163
Participants	6	(*a*) Give the eligibility criteria, and the sources and methods of case ascertainment and control selection. Give the rationale for the choice of cases and controls lines 91-97
		(*b*) For matched studies, give matching criteria and the number of controls per case lines 91-97
Variables	7	Clearly define all outcomes, exposures, predictors, potential confounders, and effect modifiers. Give diagnostic criteria, if applicable lines 155-163
Data sources/ measurement	8*	For each variable of interest, give sources of data and details of methods of assessment (measurement). Describe comparability of assessment methods if there is more than one group lines 106-163
Bias	9	Describe any efforts to address potential sources of bias not applicable
Study size	10	Explain how the study size was arrived at not applicable but post hoc power analyses (power and effect size) were calculated in lines 297-300
Quantitative variables	11	Explain how quantitative variables were handled in the analyses. If applicable, describe which groupings were chosen and why lines 124-127
Statistical methods	12	(*a*) Describe all statistical methods, including those used to control for confounding lines 165-177
		(*b*) Describe any methods used to examine subgroups and interactions lines 171-174
		(*c*) Explain how missing data were addressed no missing data in this study
		(*d*) If applicable, explain how matching of cases and controls was addressed not applicable
		(*e*) Describe any sensitivity analyses lines 297300
**Results**		
Participants	13*	(a) Report numbers of individuals at each stage of study—eg numbers potentially eligible, examined for eligibility, confirmed eligible, included in the study, completing follow-up, and analysed only one time measurement
		(b) Give reasons for non-participation at each stage only one time measurement
		(c) Consider use of a flow diagram only one time measurement
Descriptive data	14*	(a) Give characteristics of study participants (eg demographic, clinical, social) and information on exposures and potential confounders lines 181-182 (table 1)
		(b) Indicate number of participants with missing data for each variable of interest

		table 1 (no missing data in this study)
Outcome data	15*	Report numbers in each exposure category, or summary measures of exposure lines 182-196(table 2, table 3 and figure 3)
Main results	16	(*a*) Give unadjusted estimates and, if applicable, confounder-adjusted estimates and their precision (eg, 95% confidence interval). Make clear which confounders were adjusted for and why they were included lines 184-196
		(*b*) Report category boundaries when continuous variables were categorized not applicable
		(*c*) If relevant, consider translating estimates of relative risk into absolute risk for a meaningful time period not applicable

Other analyses	17	Report other analyses done—eg analyses of subgroups and interactions, and sensitivity analyses lines 184-196; lines 297-300
**Discussion**		
Key results	18	Summarise key results with reference to study objectives lines 231-296
Limitations	19	Discuss limitations of the study, taking into account sources of potential bias or imprecision. Discuss both direction and magnitude of any potential bias lines 302-316
Interpretation	20	Give a cautious overall interpretation of results considering objectives, limitations, multiplicity of analyses, results from similar studies, and other relevant evidence lines 320-326
Generalisability	21	Discuss the generalisability (external validity) of the study results lines 284-296; lines 320-326
**Other information**		
Funding	22	Give the source of funding and the role of the funders for the present study and, if applicable, for the original study on which the present article is based we provide it via PLOS ONE submission system.

*Give information separately for cases and controls.

Note: An Explanation and Elaboration article discusses each checklist item and gives methodological background and published examples of transparent reporting. The STROBE checklist is best used in conjunction with this article (freely available on the Web sites of PLoS Medicine at http://www.plosmedicine.org/, Annals of Internal Medicine at http://www.annals.org/, and Epidemiology at http://www.epidem.com/). Information on the STROBE Initiative is available at http://www.strobe-statement.org.
